# Psychological Distress Among Adolescent Students Following the April 25, 2015 Massive Earthquake in Nepal

**DOI:** 10.7759/cureus.4809

**Published:** 2019-06-03

**Authors:** Srijan Adhikari, Abhishek Thakur, Cristina Pratt, Richard Feinn, Wendy Sewack, David Hill

**Affiliations:** 1 Neurosurgery, Frank H. Netter MD School of Medicine, North Haven, USA; 2 Internal Medicine, Frank H. Netter MD School of Medicine, North Haven, USA; 3 Statistics, Frank H. Netter MD School of Medicine, North Haven, USA; 4 Global Public Health, Frank H. Netter MD School of Medicine, North Haven, USA

**Keywords:** psychological distress, nepal, earthquake, adolescent, 2015, depression, anxiety

## Abstract

Introduction

A massive earthquake on April 25, 2015, resulted in physical and emotional devastation in Nepal. This study aims to determine the prevalence of psychological distress among adolescents in Kathmandu and Sindhupalchowk districts within Nepal one year after the earthquake.

Methods

The Brief Symptoms Inventory tool was used to measure the level of psychological distress. The participants were students of four randomly selected schools from both districts. Surveys were conducted involving 200 students aged 13 to 17 years. Participants had diverse socioeconomic and cultural backgrounds.

Results

The prevalence of clinical threshold varied from 10% to 50% depending on each of the nine symptoms scales. Between the two districts, there was a statically significant difference in the prevalence of major psychological distresses. Sindhupalchowk had a higher percentage of students meeting the clinical threshold in each of the nine symptom scales than Kathmandu. Female students tended to have higher symptoms levels than male students.

Conclusion

The prevalence of psychological distress among adolescents living in areas of large impact is greater compared to the prevalence of psychological distress in adolescents living in less impacted areas. Given the current literature with respect to adolescent psychology in Nepal, more studies must be done to assess the level of distress in other regions of the country.

## Introduction

On April 25, 2015, a massive earthquake struck Nepal, causing widespread devastation. A total of 8,856 individuals lost their lives, and 22,309 people were severely injured. Hundreds of thousands of buildings were either damaged or destroyed, taking a massive toll on the infrastructure of Nepal [1].

After a natural disaster such as an earthquake, many factors influence the prevalence of psychological distress in a population. The prevalence of psychological distress may differ based on the severity of the exposure and location [2]. Natural disasters, like earthquakes, can have a significant impact on human psychology that can last for years [3]. After an earthquake, high rates of psychological distress such as anxiety and depression are common, especially among vulnerable populations such as those in low-resource settings and children/adolescents [4]. The psychological trauma from natural disasters can have a significant impact on the development of children and adolescents and may lead to more psychological disorders in the future [5]. Studies have shown high rates of post-traumatic stress disorder (PTSD), anxiety, and depression in children and adolescents following earthquakes. Studies have also shown that psychological distress is significantly underestimated in children and adolescents [6]. Thus, it is imperative to conduct research assessing psychological distress in this age group to implement interventions to reduce such burdens, especially in countries like Nepal, where these evaluations are not performed on a regular basis.

In this study, we aim to compare the rate of psychological distress in adolescents in two Nepalese districts, each district having experienced a different level of earthquake severity in 2015. In Western Nepal, the earthquake had a much larger impact in the district of Sindhupalchowk compared to the Kathmandu district. We aimed to determine the prevalence of psychological distress among adolescents in Kathmandu and Sindhupalchowk districts. Additionally, we aimed to determine whether rates of psychological distress were higher in school districts with lower resources such as public schools compared to private schools.

We hypothesized that, while the earthquake left a marked impact on all adolescents in the study, students who live in the areas where the destruction was greater will have more severe psychological distress than those living in areas with less destruction. Additionally, students who are from a lower socioeconomic background will have higher incidences of psychological distress than those with greater socioeconomic resources.

## Materials and methods

After gaining approval from the Nepal Government Department of Education to conduct the study, we obtained the lists of schools from Kathmandu district and Sindhupalchowk district from the Ministry of Education. We also received institutional review board approval from Nepal Health Research Council and Frank H. Netter School of Medicine, Quinnipiac University. We randomly selected one public school and one private school from each district. The study included 200 participating students from four different schools (Table 1).

**Table 1:** Participant distribution by district, school type, and gender.

School Type	District	Male Students (n)	Female Students (n)	Total (N)
Public	Kathmandu	47	29	76
Private	Kathmandu	26	15	41
Public	Sindhupalchowk	21	16	37
Private	Sindhupalchowk	16	30	46
Total		110	90	200

Students from grade eight to 10 aged 13 to 17 years were included in the study. Interested students were given a consent form in the Nepali language one day prior to administering the survey so that they had enough time to discuss participating in the study with their parents or legal guardians. The students provided informed consent to participate, along with signed consent from parents and legal guardians. The Brief Symptoms Inventory (BSI) instrument was used to assess levels of psychological distress [7]. The BSI tool constitutes 53 different questions to evaluate nine different symptoms scales: SOM, Somatization; O-C, Obsessive-Compulsive; I-S, Interpersonal Sensitivity; DEP, Depression; ANX, Anxiety; HOS, Hostility; PHOB, Phobic Anxiety; PAR, Paranoid Ideation; and PSY; Psychoticism. BIS has a five-point scale to determine symptom severity. Students were shown a picture of five different glasses with one being completely empty and four with a gradual increase in water content; the final glass was completely full. We used the picture as an analogy for the students to rate the severity of their distress. The survey was administrated in specific classrooms according to school year levels. The survey was in English. However, the instructor translated it verbally to Nepali for each question. Students were given adequate time to provide answers before moving to the next question. The same instructor was used in every classroom for both districts.

After the surveys were completed, data from individual students were scored using BSI scoring software. Individual scoring profiles were generated and further analyzed using IBM SPSS Statistics for Windows, Version 24.0 (IBM Corp., Armonk, NY). Multivariate analysis of variance (MANOVA) was used to compare districts, gender, and school type on the combined nine symptom subscales of the BSI. This was followed by univariate analysis of variance to identify which specific subscales differed. A model including all three factors of district, gender, and school type together did not result in statistically significant interactions, and thus, only main effect models are presented. Statistical significance was set at an alpha level of .05.

## Results

**Figure 1:**
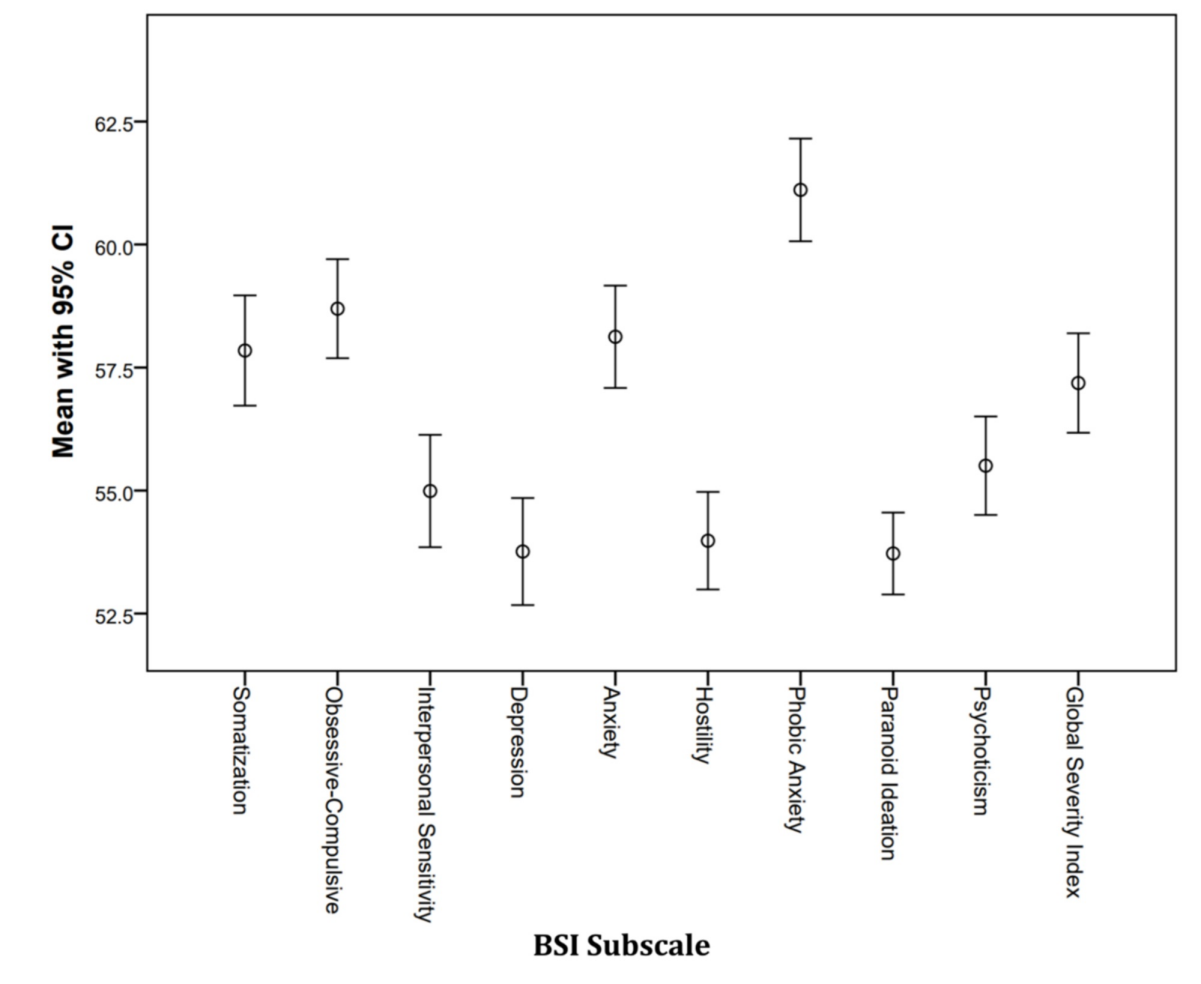
Psychological distress score by BSI subscale. Error bars indicate the mean score (95% CI). CI: Confidence interval; BSI: Brief Symptoms Inventory.

Table 1 shows the distribution of participants and gender with respect to their school's location in a specific district. The average BSI subscale scores were all above the normalized mean of 50, ranging from 53.7 (Paranoid Ideation) to 61.1 (Phobic Anxiety). Figure 1 shows the mean score with a 95% confidence interval for each subscale and the combined Global Severity Index score. The lower error bars all exceed 50, indicating greater distress among the 200 Nepalese students compared to reference levels developed in America.

**Figure 2:**
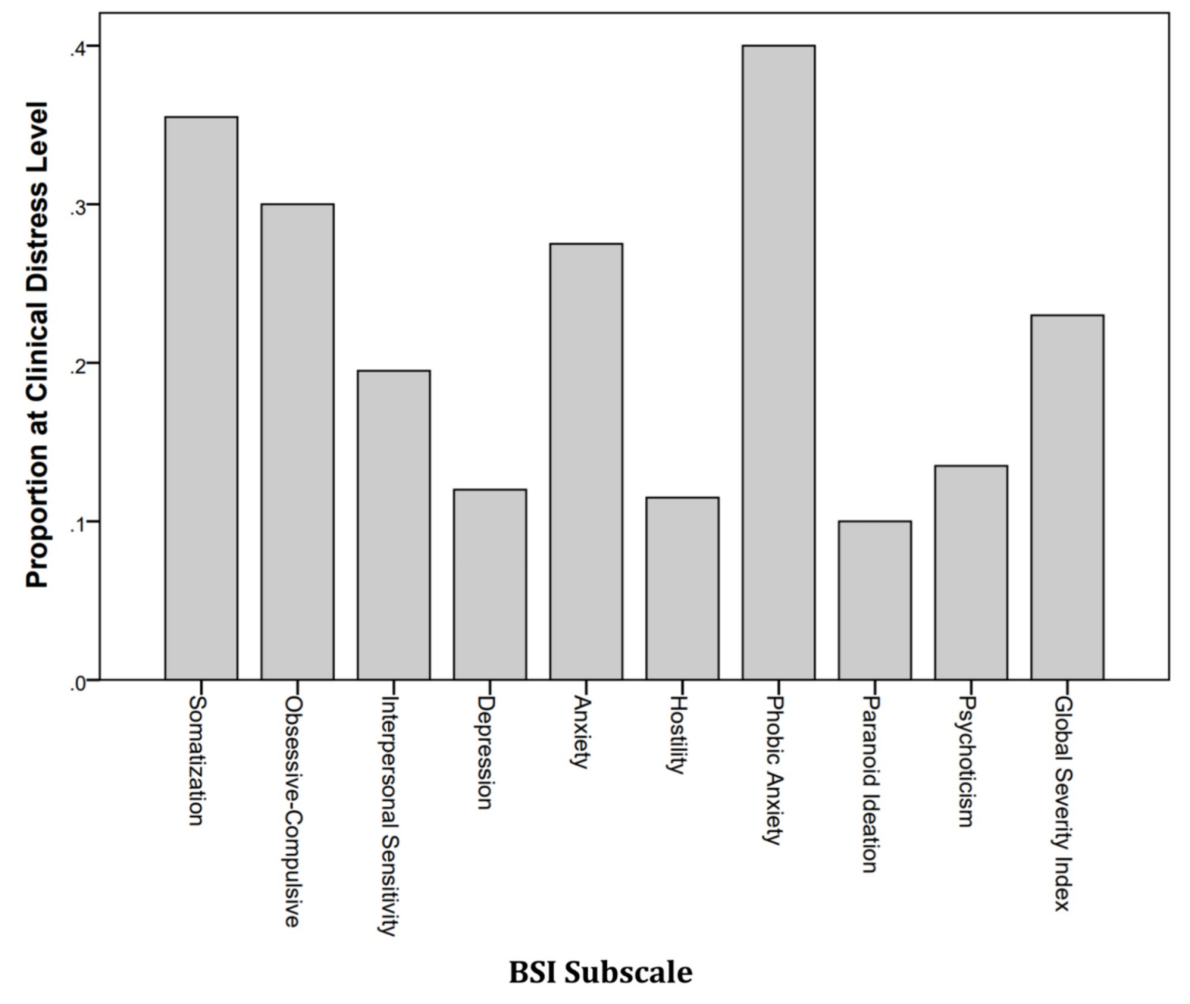
Proportion of students with distress level meeting clinical concern. BSI: Brief Symptoms Inventory.

Figure 2 shows the proportion of students who met the criteria of clinical concern (i.e., a score of 63 or more). This varied from 10% (Paranoid Ideation) to 40% (Phobic Anxiety) with 23% for overall Global Severity and is much higher than the 2% to 3% reference range.

District

**Figure 3:**
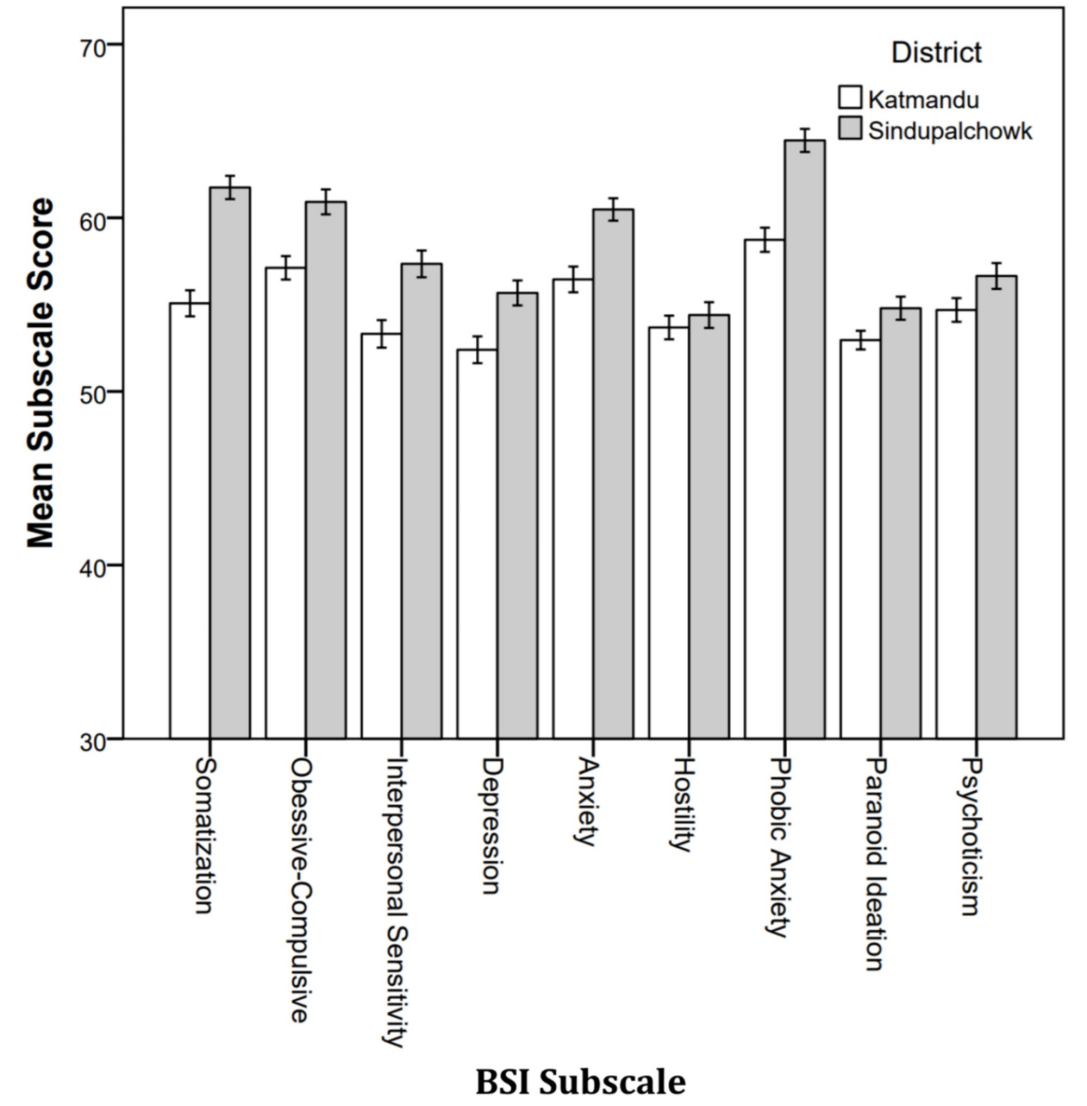
Mean BSI subscale score by district. BSI: Brief Symptoms Inventory.

The MANOVA comparing Sindhupalchowk to Kathmandu resulted in a significant difference (Wilks’ λ = .764, F9, 190, p < .001). Figure 3 shows the mean subscale score by district. Sindhupalchowk students reported higher levels of distress on every subscale. The difference was statistically significant for every subscale except Hostility (p = .486) and Psychoticism (p = .057).

**Figure 4:**
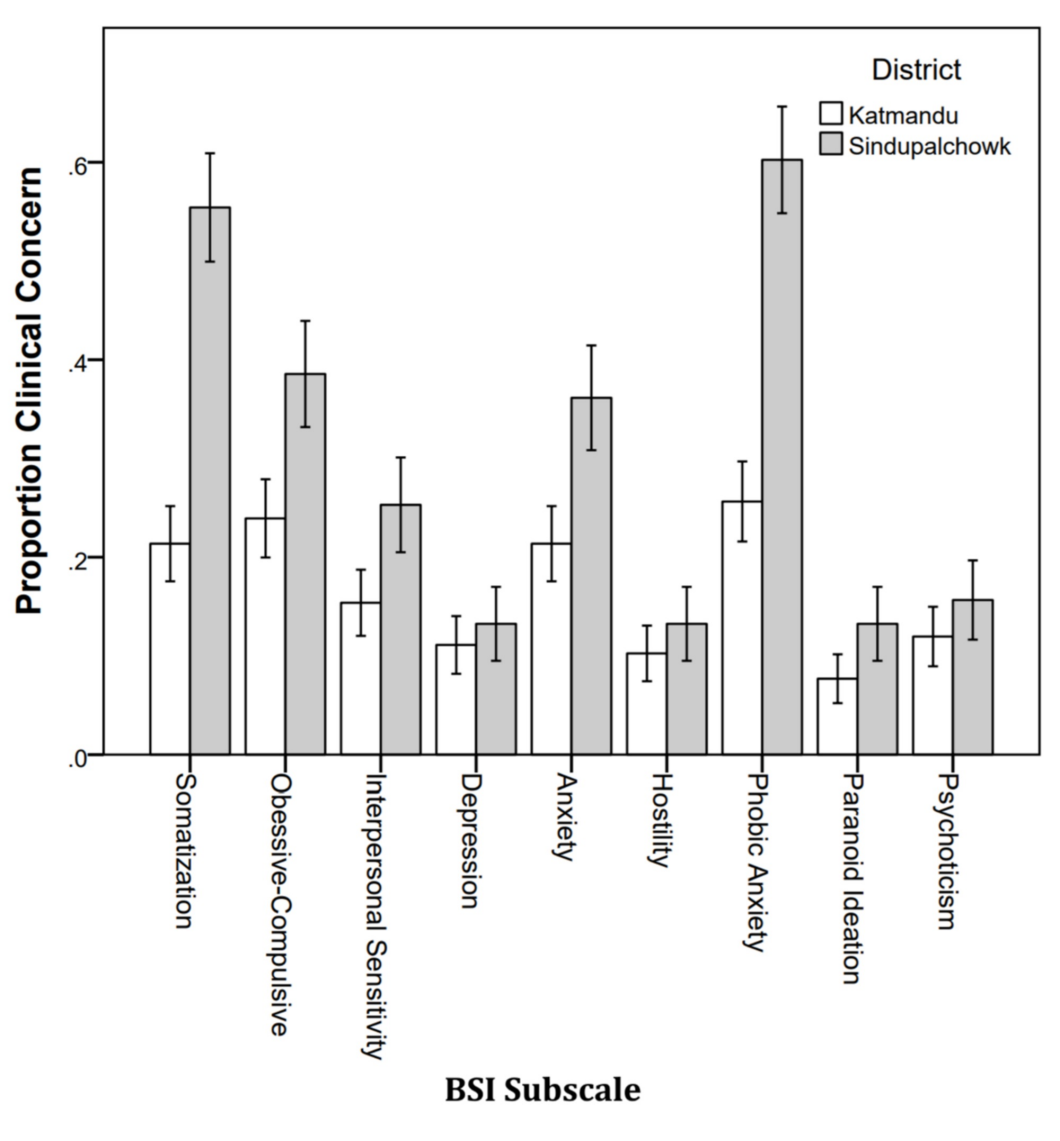
Proportion of students with clinical concern by district. BSI: Brief Symptoms Inventory.

Figure 4 shows the proportions of students with clinical concern by district. More students from Sindhupalchowk were in the clinical concerned range than Kathmandu and differed significantly on somatization (p < .001), obsessive-compulsive (p = .026), anxiety (p = .021), and phobic anxiety (p < .001).

Gender

**Figure 5:**
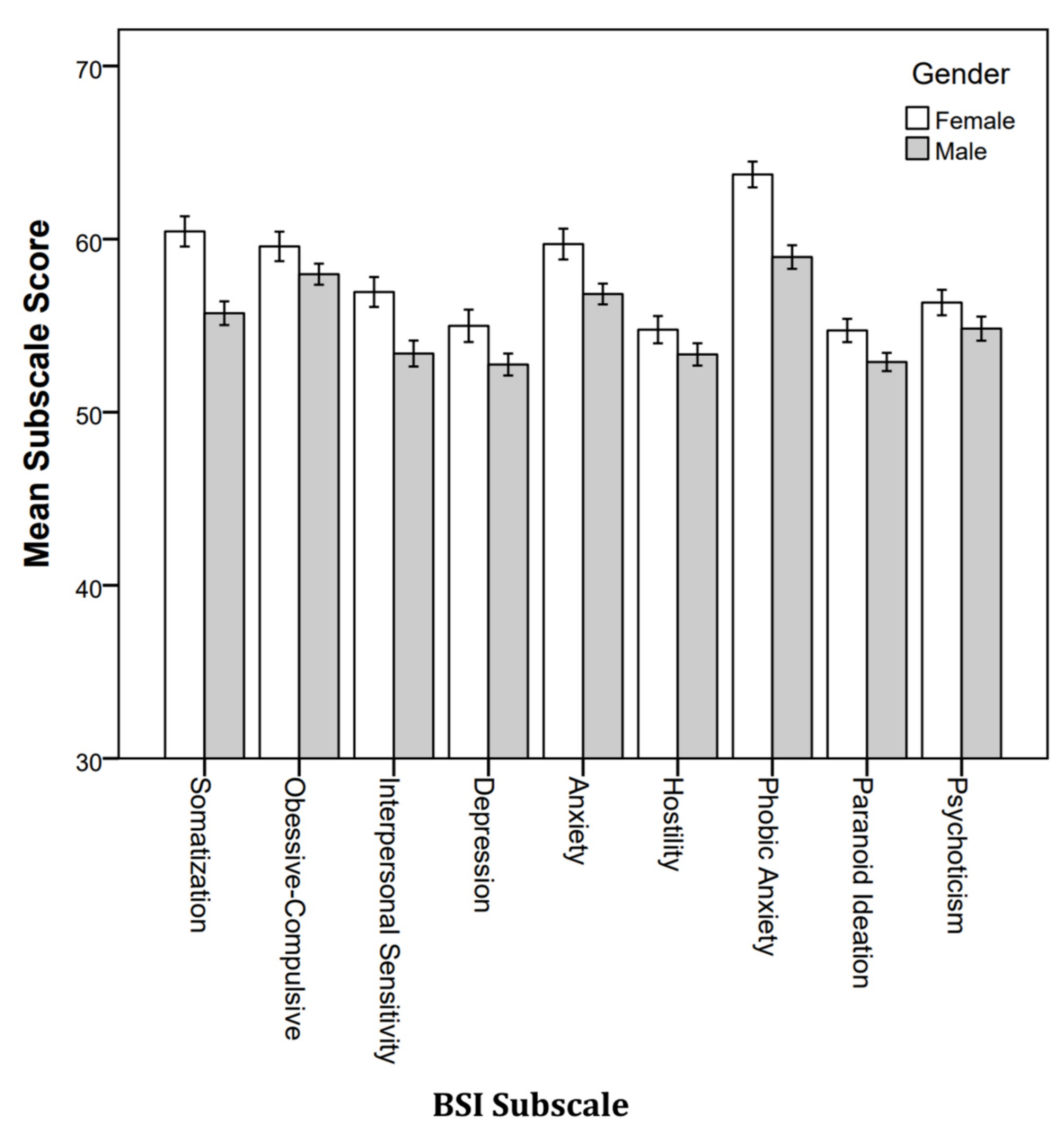
Mean BSI subscale score by gender. BSI: Brief Symptoms Inventory.

The MANOVA comparing the genders resulted in a significant difference (Wilks’ λ = .849, F9, 190, p < .001). Figure 5 shows the mean subscale score by gender. Female students reported higher levels of distress on every subscale. The difference was statistically significant on somatization (p < .001), interpersonal sensitivity (p = .002), depression (p = .044), anxiety (p = .006), phobic anxiety (p < .001), and paranoid ideation (p = .031).

**Figure 6:**
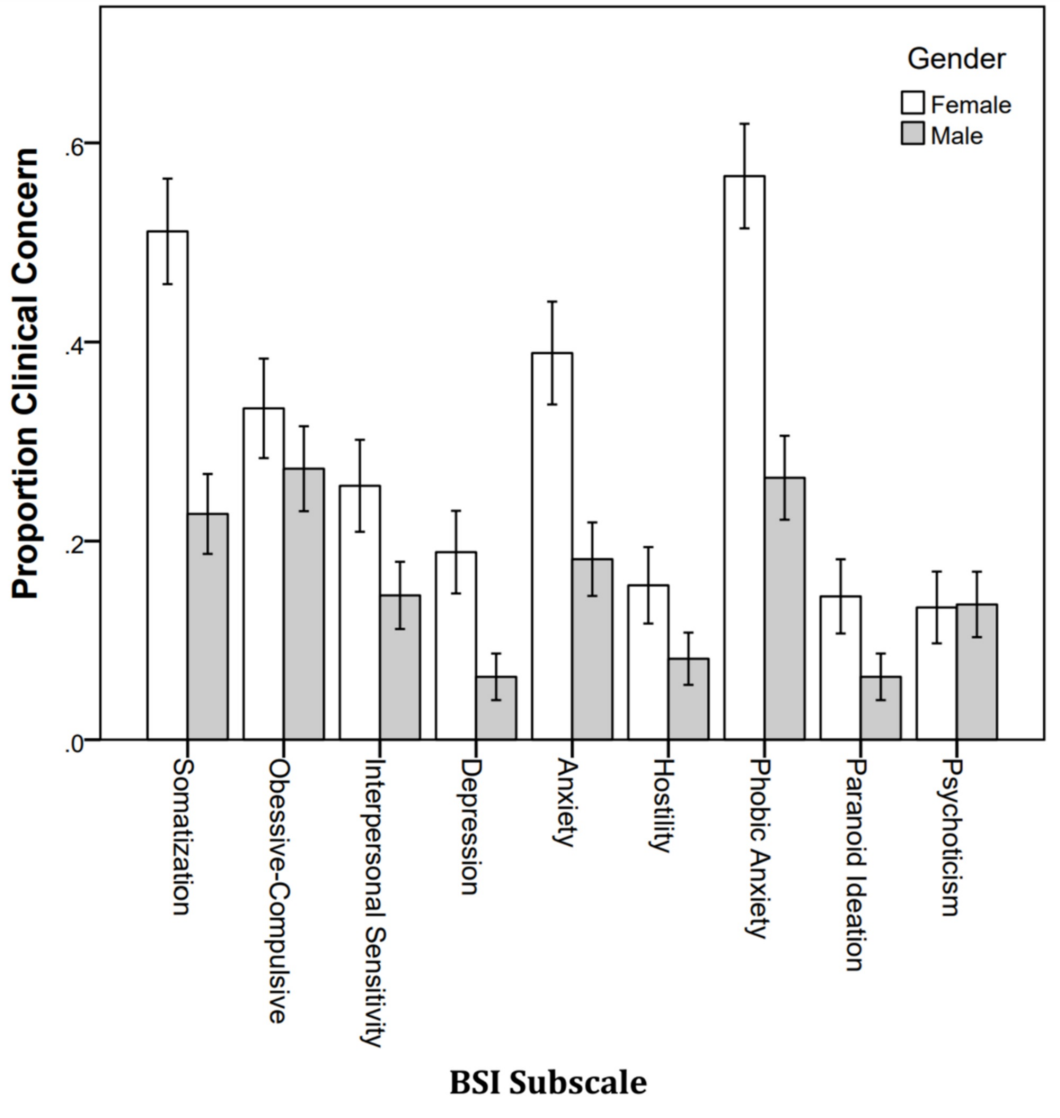
Proportion of students with clinical concern by gender. BSI: Brief Symptoms Inventory.

Figure 6 shows the proportion of students with clinical concerns by gender. More female students were in the clinical concerned range than male students, and this differed significantly on somatization (p < .001), depression (p = .007), anxiety (p = .001), and phobic anxiety (p < .001).

School type

**Figure 7:**
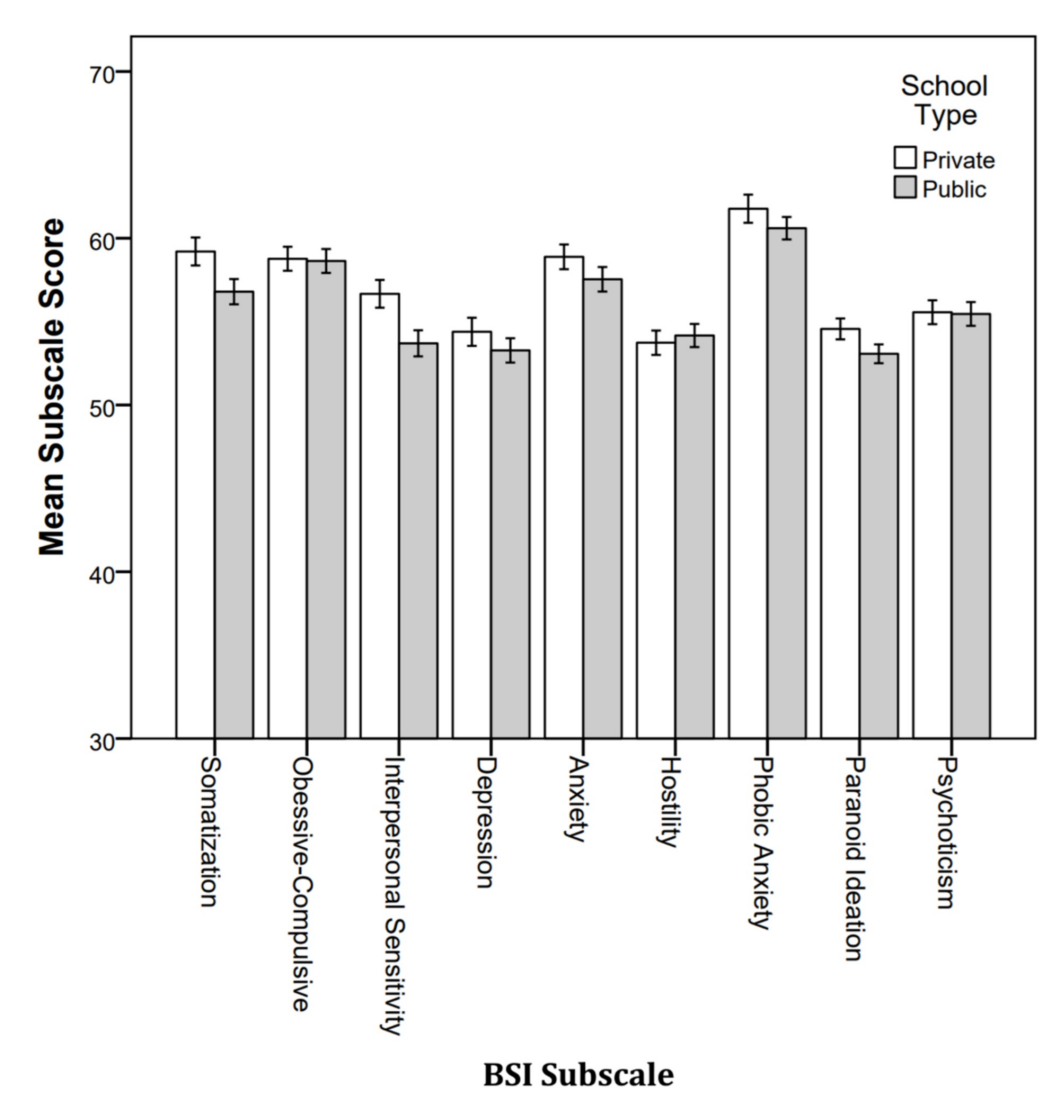
Mean BSI subscale score by gender. BSI: Brief Symptoms Inventory.

The MANOVA comparing private versus public schools did not result in a significant effect (Wilks’ λ = .913, F9, 190, p = .061). Figure 7 shows the mean subscale scores by school type, and overall, the scores are similar between schools. However, there was a significant difference between school types in the clinical concern range (Wilks’ λ = .895, F9, 190, p = .011).

**Figure 8:**
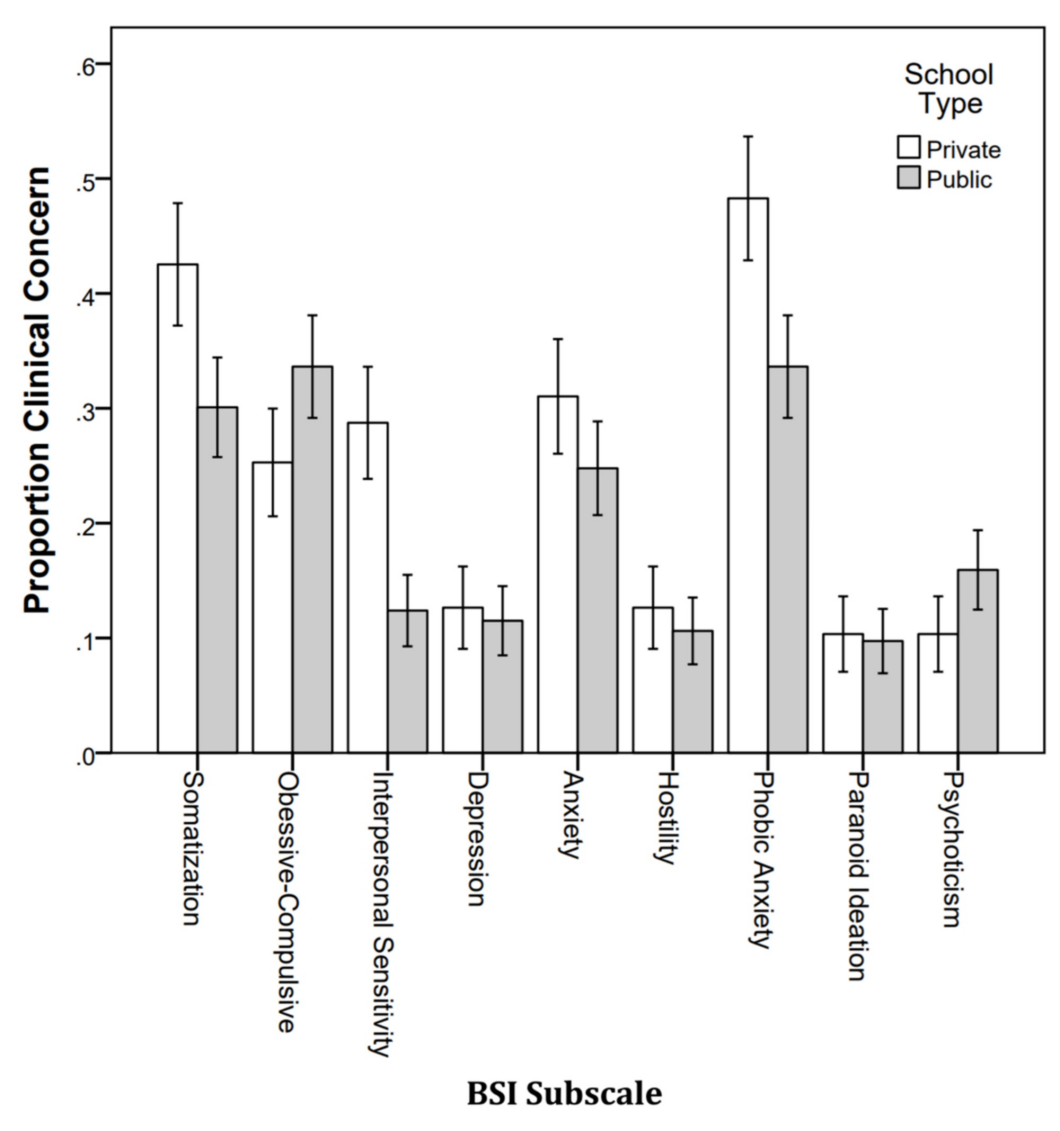
Proportion of students with clinical concern by school type. BSI: Brief Symptoms Inventory.

Figure 8 shows the proportion of students with clinical concern by school type. Students from private schools had a higher proportion of interpersonal sensitivity (p = .004), and phobic anxiety (p = .036).

## Discussion

The result of this study not only further solidifies the results from previous studies done among the victims of natural disasters but also confirms that the impact of an earthquake on psychological distress lingers more than 15 months after the event [2]. Overall, we found that students from both districts scored higher than the normalized mean of 50. This could be explained by the fact that the earthquake affected the whole country. Though the Kathmandu district had a lower impact than Sindhupalchowk, many students still felt the quake and had to live outside their homes for weeks.

When we compared the two districts, the students from Sindhupalchowk reported a higher level of distress in all the subscales. In addition, more students from Sindhupalchowk were in the clinical concerned range than students from Kathmandu, which supports our hypothesis. However, a substantial number of students (>60%) reported higher distress in at least one subscale. This result alone warrants further interventional studies to help reduce the distress level.

Our findings suggest that more female students in both districts reported a higher level of psychological distress than males. When considering gender, many studies have shown that female children and adolescents suffering from natural disasters are more susceptible to psychological distress like PTSD and anxiety when compared to male children [8]. Few studies contradict this finding and show that there is no statistically significant difference among the genders [9]. Even though there is no concrete evidence, cultural values, and gender roles in Nepal can also be factors. Further studies must be done to determine the exact reason for the variance seen in this study.

When we looked at the results between public and private schools, we found no significant difference. We chose public and private schools because, in general, people in Nepal from lower socioeconomic background send their children to public schools. Previous studies have shown that individuals with low socioeconomic status (SES) tend to have a higher prevalence of psychological distress [10]. Sindhupalchowk is among the poorer districts of Nepal, so students from both public and private schools can be of similar SES, explaining the lack of difference. In addition, the entire country suffered from an impactful earthquake, which could mask the subtle changes in psychological distressed due to SES; however, there is no solid evidence for either of these explanations. While this study intends to compare psychological distress after the earthquake between these groups of potentially varying SES, it is important to keep in mind that there may have been a difference in distress present even prior to the earthquake. As stated above, SES is itself a determinant of distress, and the earthquake may have exacerbated the effect to varying degrees.

The findings of our study have implications for policymaking for the ministry of health and education in Nepal. Thus far, there are not many studies in Nepal on reducing the burden of psychological distress among children and adolescents. Our findings indicate that an intervention is warranted. Students in Nepal are living their lives in severe psychological distress when they should be studying and enjoying childhood. With that in mind, we have initiated a follow-up interventional study to determine the best ways to reduce the psychological burden in Nepali children that will include the use of yoga and aerobic exercise as potential interventions to reduce psychological distress [11].

Limitations

Our study had several limitations. We had no standard instrument in Nepalese language to collect our data; however, a bilingual survey administrator who could clearly explain the details in the questionnaire was helpful. We had no information about the psychological status of the students before the earthquake, which limited our ability to compare our results from the past. However, we designed our experiment as a comparative study between two districts to mitigate this. We did not collect data on different psychological stress risk factors such as the death of family members, serious physical trauma, and supportive factors. Finally, our study would have benefited from a larger sample size.

## Conclusions

This study examined the psychological distress among adolescents in two districts of Nepal after the massive earthquake of 2015. The results showed adolescents living in areas of large impact (i.e., the Sindhupalchowk district) suffered greater psychological distress than to those in less impacted regions, and that female participants experienced more distress than male ones. Given the current literature with respect to adolescent psychology in Nepal as well as the findings of this study, future studies must be done to truly assess the level of distress in other regions of the country to allow for policy-making and public health interventions.
